# AG‐exclusion zone revisited: Lessons to learn from 91 intronic NF1 3′ splice site mutations outside the canonical AG‐dinucleotides

**DOI:** 10.1002/humu.24005

**Published:** 2020-03-11

**Authors:** Katharina Wimmer, Esther Schamschula, Annekatrin Wernstedt, Pia Traunfellner, Albert Amberger, Johannes Zschocke, Peter Kroisel, Yunjia Chen, Tom Callens, Ludwine Messiaen

**Affiliations:** ^1^ Institute of Human Genetics, Department of Genetics and Pharmacology Medical University of Innsbruck Innsbruck Austria; ^2^ Diagnostic & Research Institute of Human Genetics, Diagnostic & Research Center for Molecular BioMedicine Medical University of Graz Graz Austria; ^3^ Department of Genetics University of Alabama at Birmingham Birmingham Alabama

**Keywords:** 3′ splice site, AG exclusion zone, *NF1* gene, noncanonical splice mutation, variant of unknown significance

## Abstract

Uncovering frequent motives of action by which variants impair 3′ splice site (3′ss) recognition and selection is essential to improve our understanding of this complex process. Through several mini‐gene experiments, we demonstrate that the pyrimidine (Y) to purine (R) transversion NM_000267.3(*NF1*):c.1722‐11T>G, although expected to weaken the polypyrimidine tract, causes exon skipping primarily by introducing a novel AG in the AG‐exclusion zone (AGEZ) between the authentic 3′ss AG and the branch point. Evaluation of 90 additional noncanonical intronic *NF1* 3′ss mutations confirmed that 63% of all mutations and 89% (49/55) of the single‐nucleotide variants upstream of positions ‐3 interrupt the AGEZ. Of these AGEZ‐interrupting mutations, 24/49 lead to exon skipping suggesting that absence of AG in this region is necessary for accurate 3′ss selection already in the initial steps of splicing. The analysis of 91 noncanonical *NF1* 3′ss mutations also shows that 90% either introduce a novel AG in the AGEZ, cause a Y>R transversion at position ‐3 or remove ≥2 Ys in the AGEZ. We confirm in a validation cohort that these three motives distinguish spliceogenic from splice‐neutral variants with 85% accuracy and, therefore, are generally applicable to select among variants of unknown significance those likely to affect splicing.

## Graphical abstract

Our analysis of the largest number (*n* = 91) of noncanonical 3′ splice site mutations found in a single gene by using an unbiased RNA‐based mutation analysis approach shows that interruption of the AG‐exclusion zone is the major motive of action of these mutations. In a validation cohort we show that this and two additionally identified motives of action distinguish spliceogenic from splice‐neutral variants with 85% accuracy. Therefore, these three motives are generally applicable to select among intronic variants of unknown significance, even when located upstream of position ‐12, those likely to affect splicing.

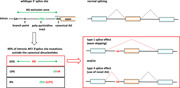

## 1 INTRODUCTION

Accurate splicing of pre‐mRNA is essential for the production of functional proteins from the vast majority of all human genes. As a consequence sequence variants that affect splicing precision play an important role in hereditary disorders (Baralle & Buratti, 2017; Wang & Cooper, 2007). The recognition and selection of the proper 5′ (donor) splice site (5′ss) and 3′ (acceptor) splice site (3′ss), defining the borders of introns, are paramount for accurate splicing. Dissecting the specific mechanisms by which individual sequence variants interfere with splice site recognition and selection leads to a better understanding of these complex and still poorly understood processes.

Several studies from our and other groups have shown that for the *NF1* gene, which is mutated in individuals with neurofibromatosis type 1 (NF1), the proportion of pathogenic variants that alter splicing, for simplicity named splice mutations throughout the paper, is among the highest found in human disease genes (Ars et al., 2000; Messiaen et al., 2000; Wimmer et al., 2007). About two‐thirds of these splice mutations are located outside the canonical GT and AG nucleotides (Messiaen & Wimmer, 2008). Several reasons may in concert account for these observations. First, a high proportion of splice mutations may be intrinsic to the structure of this large gene which is composed of 57 constitutively spliced exons and, hence, has a high number of splice sites that may be altered by mutations. Second, even “leaky” *NF1* splice mutations, that is splice mutations leading to a splice effect only in a proportion of, but not in all, transcripts from this allele, may result in an attenuated but still clinically recognizable NF1 phenotype. This has been observed in a number of patients with typical cutaneous NF1 features such as multiple café au lait maculae (CALM) and cutaneous neurofibromas (see e.g., Fernandez‐Rodriguez et al., 2011), including a case that will be discussed in this report. This phenomenon may differentiate *NF1* from other genes associated with less specific symptoms, for example an increased cancer risk, that may not be recognized when present in an attenuated/mild form. In other words, the high frequency (compared to other genes) of noncanonical *NF1* splice mutations observed may in part result from ascertainment bias, since mild NF1 phenotypes resulting from “leaky” splice mutations may come more frequently to clinical attention than attenuated phenotypes in other syndromes. Finally, and probably most importantly, splice defects are more effectively uncovered in this gene than in other genes, since our and a few others laboratories systematically apply RNA‐based protocols as the first line mutation analysis assay. In our laboratories, direct complementary DNA (cDNA) sequencing of the entire coding sequence starting with RNA extracted from short‐term lymphocyte cultures treated with puromycin to prevent nonsense‐mediated RNA decay, proved to be highly sensitive also for the detection of noncanonical splice mutations (Messiaen & Wimmer, 2008).

Having applied this mutation analysis strategy for roughly two decades, we have identified a large number of noncanonical *NF1* splice mutations fully characterized at the transcript level. They provide now a unique data set to gain insights into the mechanisms of action of noncanonical splice mutations, which in turn will help to establish the processes by which splice sites are recognized and selected. Furthermore, a better understanding of the mechanisms by which noncanonical splice mutations affect splicing will have immediate implications for molecular genetic diagnostics of NF1 and beyond, as it may lead to improved algorithms to stratify which gene variants found by sequencing of genomic DNA (gDNA) are likely to have an impact on splicing and should, therefore, be further analyzed at the RNA level. This will, in turn, reduce the number of variants of unknown significance (VUS) and improve the diagnostic data return from massive parallel gDNA sequencing.

In this study, we focused on mechanisms of action of noncanonical intronic 3′ss mutations. During the initial steps of splicing, degenerated sequence motives at the splice sites are bound by small nuclear ribonucleoproteins snRNPs. While the 5′ss is primarily defined by a 9‐nuclotides (nts) sequence motive that base‐pairs with the RNA moiety of the U1 snRNP, the sequence elements defining the 3′ end of an intron are more complex. They consist of three highly degenerate sequence motives: the branch point (BP), the polypyrimidine tract (PPT), and the 3′ss invariant AG‐dinucleotide which is most frequently (96% of 3′ss) preceded by a pyrimidine residue (Y). In the initial steps of 3′ss recognition (assembly of the complex E) the branchpoint sequence (BPS) is bound to the branch point binding protein SF1/BBP which later is replaced by the U2 snRNP (complex A). The 3′ss AG and the PPT are recognized and bound by the 35‐ and 65‐kDa subunits of the U2 snRNP Auxiliary Factor (U2AF), respectively. U2AF65 forms a stable heterodimer with U2AF35 interacts with the BPS bound SF1/BBP and helps to recruit the SF1/BBP‐replacing U2 snRNP to the BPS (Wahl, Will, & Luhrmann, 2009 and references cited therein).

An active spliceosome is formed after the recruitment of further snRNPs and several conformational rearrangements. The first step of splicing involves a hydrophilic attack by the 2′‐OH of the BP adenosine on the 5′ss, leading to the formation of the 5′‐exon and the intron lariat intermediate. In the second step, the 3′‐OH of the 5′‐exon attacks the 3′ss, leading to exon joining and intron excision (Wahl et al., 2009). For accurate splicing, the spliceosome must locate the AG‐dinucleotide that defines the 3′ss in this second catalytic step. Several lines of evidence show that the spliceosome has a strong preference for use of the first AG‐dinucleotide downstream of the BP (Anderson & Moore, 1997; Chua & Reed, 2001; Krainer & Maniatis, 1985; Umen & Guthrie, 1995). As a consequence the sequence between the BP and the proper 3′ss AG is devoid of AGs and, therefore, is called an AG‐exclusion zone (AGEZ; Gooding et al., 2006). The AGEZ mainly found appreciation as a tool to search for potential (distant) BP (Gooding et al., 2006).

Here we show that interruption of the AGEZ by a mutation‐created AG is the most frequent motive of action of noncanonical intronic 3′ss mutations. In two‐thirds of the mutations, this leads to using the mutation‐created AG instead of the authentic 3′ss, but in nearly half of the mutations, this leads also to exon skipping in a proportion or in all transcripts of the mutated allele. This suggests that the absence of AG dinucleotides in this region is not only necessary to ensure the selection of the authentic 3′ss during catalytic Step II of splicing, but also for accurate recognition and selection of the authentic 3′ss in the initial steps of splicing.

We further show that nearly 90% of all bona fide splice mutations follow this or two additional action motives. These three motives will be useful to select for further transcript analysis that intronic VUS found at a gDNA level that are most likely to have a splice effect.

## 2 MATERIAL AND METHODS

### 2.1 Patients

Between 1998 and 2018, in 8,690 unrelated patients sent for clinical *NF1* testing to two centers, that is the Medical Genomics Laboratory at the University of Alabama in Birmingham (UAB) and the Institute of Human Genetics, Medical University Innsbruck (MUI), and *NF1* reportable variant was identified. All were analyzed by an RNA‐based protocol as the first line mutation analysis assay. The analyses were performed with informed consent from all patients or their parents.

The sporadic patient F8519 analyzed at MUI is a 28‐year‐old female. She fulfills the diagnostic criteria of NF1 with around 20–30 CALMs, bilateral Lisch nodules, at least five intradermal neurofibromas, a number of tiny nodules interpreted as cutaneous neurofibromas and an asymptomatic optic pathway glioma.

### 2.2 Identification of intronic *NF1* 3′ splice site mutations outside the canonical AG‐dinucleotides

Direct cDNA sequencing of *NF1* transcripts isolated from puromycin‐treated short‐term lymphocyte cultures is the core mutation detection method of comprehensive *NF1* mutation analysis as described by Messiaen and Wimmer (Messiaen & Wimmer, 2008; Messiaen & Wimmer, 2012; Messiaen et al., 2000). This assay reliably and effectively reveals mutation‐induced splice alterations of the *NF1* transcripts. The underlying genomic alteration is subsequently uncovered by PCR amplification and Sanger sequencing of the aberrantly spliced exon/intron. Using this approach a total of 78 different intronic 3′ss mutations outside the canonical AG‐dinucleotides were identified in 137/8250 (1.7%) unrelated and molecularly confirmed NF1 patients subjected to mutation analysis at UAB and 13/440 (2.9%) index patients analyzed at MUI (Tables S1 and S2).

Furthermore, we screened the Human Gene Mutation Database (HGMD professional; Stenson et al., 2009) and the Leiden Open Variation Database (LOVD v.2.0 Build 35, Leiden University Medical Center; https://grenada.lumc.nl/LOVD2/mendelian_genes/) for *NF1* mutations located at positions ‐50 to ‐3 of the 3′ss. Thirteen mutations with sufficient information on the associated splice effect in the original literature or deposited in the database had not been identified in our own cohort. These published mutations were also included in our analysis (Table S1). The mutations are described in accordance with the Human Genome Variation Society (http://www.hgvs.org/mutnomen) guidelines using the *NF1* mRNA sequence RefSeq NM_000267.3 as a reference with the A of the ATG start codon as position c.1. Exons/introns are numbered according to the reference sequence LRG_214 with in addition the widely known legacy numbering given in parenthesis (Messiaen & Wimmer, 2012).

### 2.3 Minigene experiments

The RTB hybrid‐minigene plasmid (Ryan & Cooper, 1996) previously provided by Dr. Thomas Cooper (Baylor College of Medicine) was used to construct minigenes containing the *NF1* exons 15 (11) and 16 (12a) with flanking intronic sequences. Wild‐type and mutant sequences were first PCR‐amplified from the genomic DNA of patient F8519 (mutation c.1722‐11A>G) and from a patient carrying mutation c.1722‐2A>G. The PCR primer pair (Table S3) was designed to amplify a 2150‐bp fragment containing the entire genomic sequence from intron 14 (10c; 230 bp upstream of exon 15) to intron 16 (12a; 212 bp downstream of exon 16). The PCR‐products from the patients’ genomic DNA contained primer‐introduced *Sal*I and *Kpn*I sites at the 5ʹ and 3ʹends, respectively, which were used to clone the PCR fragments after digestion with these restriction enzymes into the *Sal*I and *Kpn*I sites located between the internal exon 2 and the last, exon 4, of the RTB minigene. Wildtype (C1) and mutant (C2, C7) minigene constructs were selected by colony PCR and subsequent sequencing. Minigene constructs C3–C6 and C8 containing the mutations c.1722‐12A>G, c.1722‐12_1722‐11delATinsGG, c.1722‐12_1722‐11delATinsTG, c.1722‐12_1722‐11delATinsTA and c.1722‐11T>A, respectively, were generated with site‐directed mutagenesis from the minigene construct containing the wildtype intron 15 using the QuikChange®Lightning site‐directed mutagenesis kit (Agilent Technologies) according to the manufacturer's instructions. Primers for site‐directed mutagenesis are listed in (Table S3). The integrity of all final constructs was confirmed by sequencing of plasmid maxi‐preparations that were used for transient transfection in HEK293 and SH‐SY5Y cells.

For transient transfection, the widely used HEK293 cells originally derived from embryonic kidney cells were used. In addition, the neuroblastoma‐derived cell line SH‐SY5Y with a potentially different splice profile that better represents the effects of the mutations in neuronal crest‐derived cells such as Schwann cells or melanocytes were transfected. Cells of both lines were grown in six‐well plates in EMEM media (Lonza, Basel, Switzerland) containing 10% fetal bovine serum and penicillin/streptomycin/glutamine to approximately 60% confluence. They were transfected with 4 μg of plasmid DNA using Turbofect reagent (Thermo Scientific, Waltham, MA) following the manufacturer's protocol. RNA was extracted with RNeasy Mini Kit (Qiagen, Limburg, The Netherlands) 48 hr after transfection. For cDNA synthesis, 1 μg of RNA was reverse‐transcribed using random hexamers and High Capacity RNA‐to‐cDNA Kit (Applied Biosystems, Carlsbad, CA). Transfection experiments were performed at least in duplicate or triplicate. Transcripts of the hybrid RTB minigenes were PCR‐amplified using primers (RTBP4 F, RTBP4 R) located in exon 1 and 4 of the construct (Figure 2b; Table S3). The PCR products were separated on 2.0% TAE‐agarose gels. Amplification products were sequenced with primer RTBP4F.

**Figure 1:**
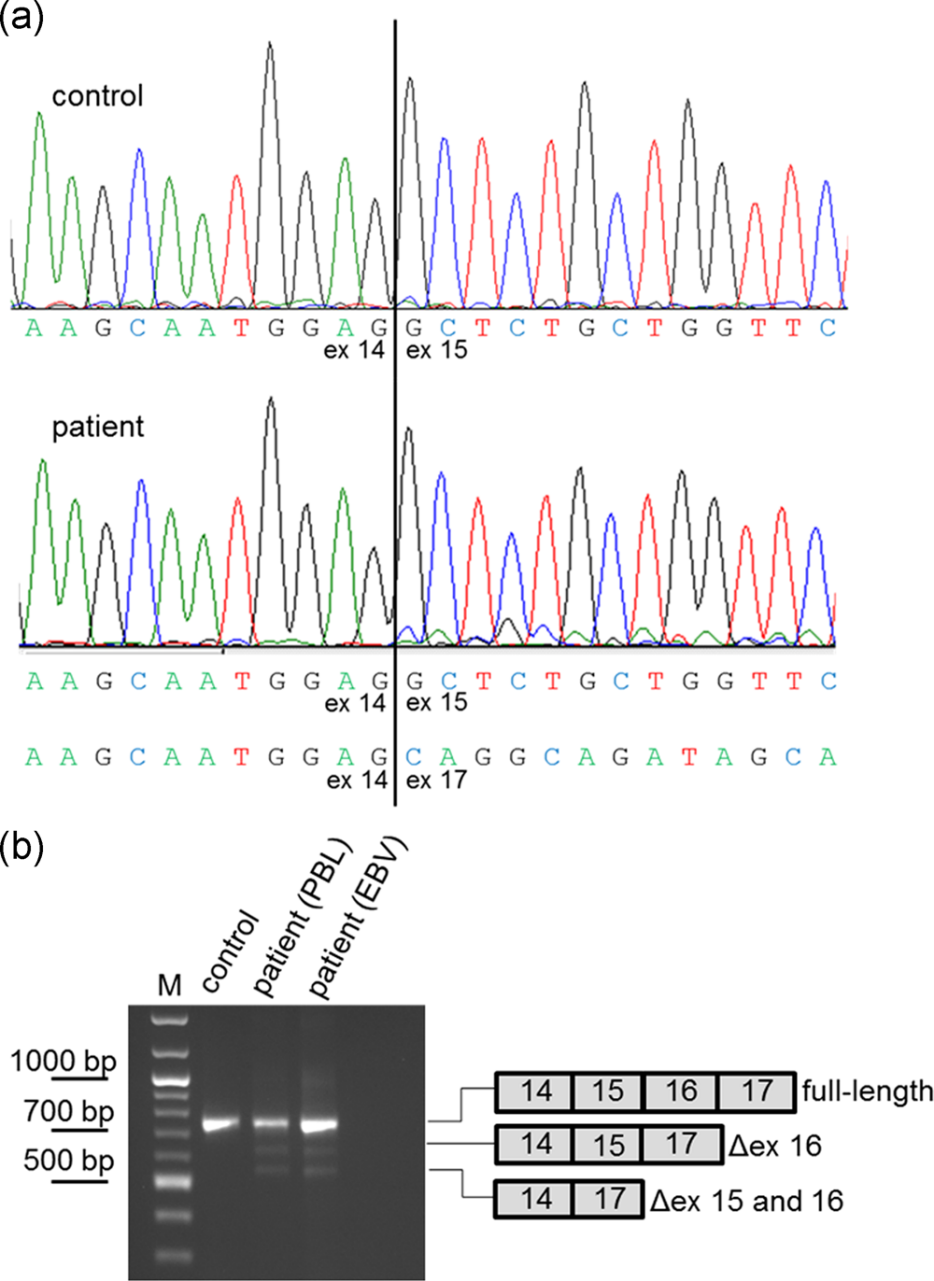
Direct complementary DNA (cDNA) sequencing of patient F8519 carrying *NF1* mutation c.1722‐11T>G reveals aberrantly spliced transcripts. (a) Sanger sequence (sense direction) of transcripts isolated from puromycin‐treated short‐term lymphocyte cultures of the patient and a non‐NF1 control individual at the border of exon 14 (ex 14) and exon 15 (ex 15). The wildtype sequence and the sequence deduced from the faint background peaks starting at the beginning of exon 15 in the patient's sequence are given below the electropherograms. This background sequence, which is absent in the control, is derived from exon 17 indicating presence of a small proportion of transcripts lacking exons 15 and 16. (b) RT‐PCR products amplified from transcripts of the patient that were isolated from a puromycin‐treated short‐term lymphocyte culture (PBL) and from EBV‐transformed lymphoblastoid cell line (EBV) also treated with puromycin before cell harvest. Two weak bands coming from a small proportion of transcripts lacking exon 16 only (∆ex 16) or exons 15 and 16 (∆ex 15 and 16) are visible. These bands are absent in the control. To allow for a better separation of the aberrant transcripts from the full‐length transcript, we used in this RT‐PCR experiment instead of our standard primers for direkt cDNA sequencing that generate a product of 1868 bp from the wildtype transcript (Messiaen & Wimmer, 2012) a primer pair that generates a shorter PCR product of the wildtype (693 bp) and aberrant (569 and 491 bp, respectively) transcripts. RT‐PCR, reverse transcriptase polymerase chain reaction

**Figure 2:**
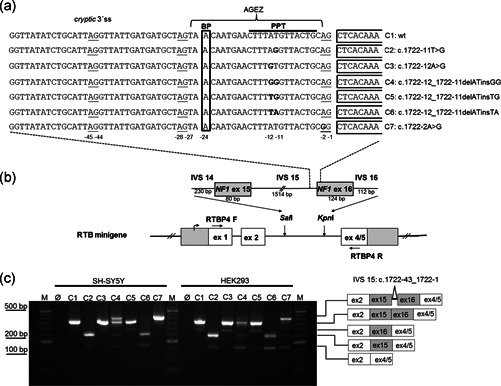
Minigene experiments confirm the splice effect of *NF1* mutation c.1722‐11T>G and reveal that the mutation acts mainly by creation of an AG‐dinucleotide within the AG exclusion zone. (a) Wildtype (wt) and mutated sequences of the intron 15 (IVS 15) 3' splice site (3'ss) which are contained in the RTB minigene, constructs C1‐C7, are shown. The first nucleotides of exon 16 (ex 16) are framed. The mutated nucleotides are in bold letters. The adenine of the predicted branch point (BP), the polypyrimidine tract (PPT) and a cryptic 3'ss are indicated. All AG‐dinucleotides in the intron are underlined and the AG exclusion zone (AGEZ) is indicated. (b) Schematic diagram of RTB minigene and the inserted *NF1* sequences containing wildtype and mutated IVS 15 and the flanking exons 15 and 16 with flanking intronic sequences. The positions of primers RTBP4F and RTBP4R used for RT‐PCR are indicated. (c) RT‐PCR results of mRNA isolated from transient transfections of SH‐SY5Y and HEK293 with minigene constructs C1‐C7. The positions of the PCR products from correctly and aberrantly spliced transcripts are indicated on the left. Use of the cryptic 3'ss leads to insertion of the last 43 nucleotides of IVS15 (c.1722‐43_1722‐1). RT‐PCR, reverse transcriptase polymerase chain reaction

### 2.4 Splice site prediction

Five in silico splice site prediction programs that can be interrogated simultaneously by the commercial Alamut Visual software (vs 10.2; Interactive Biosoftware, Rouen, France) were used to predict the effect of the *NF1* mutations and variants tested in the minigene constructs on the “strength” of splice sites, which is given by a score that is computed in each program by a different algorithm. In brief: Position weight matrices originally developed by Shapiro and Senapathy (1987) are used by the programs SpliceSiteFinder‐like and Human Splicing Finder (Desmet et al., 2009). Position weight matrices are also used by the Human Splicing Finder to score potential branch points (Desmet et al., 2009). The program MaxEntScan is based on MaxEnt, an algorithm for derivation and scoring of constrained marginal maximum entropy distributions (Yeo & Burge, 2004). The program NNSPLICE uses the machine‐learning approach of a neural networks to score splice sites (Reese, Eeckman, Kulp, & Haussler, 1997). GeneSplicer combines several splice site detection techniques, among which Markov models (Pertea, Lin, & Salzberg, 2001). The parameters were set in the Alamut Visual software (vs 10.2) to give numeric out‐put data in the range from 70 to 100 for SpliceSiteFinder‐like, 0‐16 for MaxEntScan, 0.01‐1 for NNSPLICE, 0‐21 for GeneSplicer and 65‐100 for Human Splicing Finder.

All *NF1* mutations were analyzed with the bioinformatics program Splicing Prediction in Consensus Elements (SPiCE) that combines predictions from SSF‐like and MES to generate sores that are used to predict the spliceogenicity of variants (Leman et al., 2018).

Given the severe limitations of predicting weakly conserved branch point sequences, predicted branch points were not used to define the beginning of an AGEZ. For simplicity, a mutation was considered to fall into the AGEZ when it was downstream (in 3′ direction) of the AG dinucleotide that is the first AG upstream (in 5′ direction) of the genuine 3′splice site AG.

## 3 RESULTS

### 3.1 *NF1* mutation c.1722–11T>G exerts its splice effect mainly by creating an AG‐dinucleotide within the AGEZ of the intron 15 (11) 3′ splice site

Direct cDNA sequencing of transcripts isolated from puromycin‐treated short‐term lymphocyte cultures of patient F8519 revealed a faint background sequence that is not present in control transcripts. Careful inspection of the forward and reverse sequence reads indicated that this sequence background results from two aberrantly spliced transcripts, one lacking exons 15 (11) and 16 (12a) clearly seen in the forward read (Figure 1a), and one lacking only exon 16 (12a) as deducible from the two background sequences seen in the reverse read (Figure S1). This result was confirmed in a second short term lymphocyte culture and an EBV‐transformed lymphoblastoid cell line of the patient by reverse transcriptase (RT‐)PCR amplification experiment (Figure 1b).

Based on our prior experience with other splice mutations affecting exon 16 (12a), we expected the mutation responsible for the observed splice effect in patient F8519 to be located at the intron 16 (12a) 5′ss. Mutations affecting this splice site have been reported to result in skipping of *NF1* exons 15 (11) and 16 (12a; Fang et al., 2001), a finding that we also confirmed in our own patients (unpublished data). In contrast, mutations at the AG‐dinucleotides of the 3′ss of intron 15 (11), for example, c.1722‐2A>G, lead to the use of a cryptic 3′ss located 43 nucleotides upstream of the authentic 3′ss in intron 15 (11). The small proportion of the aberrantly spliced transcripts could have been explained by mosaicism for an intron 16 (12a) 5′ss mutation. However, sequencing of exon 16 (12a) and flanking intronic sequences from genomic DNA of patient F8519 showed no evidence for the presence of a (mosaic) mutation at the 5′ss consensus sequence of the intron 16 (12a) but identified the heterozygous variant c.1722‐11T>G in the 3′ss of intron 15 (11). This variant is not recorded in dbSNP or the Genome Aggregation Database (gnomAD) nor has it been described previously in an NF1 patient. The absence of this variant in both unaffected parents further supported its pathogenicity and, hence, the implication in the observed splice effect in the patient. To confirm this assumption we tested the splice effect of the wildtype (construct C1 in Figure 2) and mutant sequences (construct C2 in Figure 2) in transcripts from minigenes transfected into SH‐SY5Y and HEK293 cells. RT‐PCR amplification of transcripts from the minigenes showed in both cell lines consistently that c.1722‐11T>G clearly leads to exon 16 (12a) or exons 16 (12a) and 15 (11) skipping, while these exons are retained in the vast majority of the transcripts from the wildtype minigene construct (Figure 2c). Remarkably, the splice effect of c.1722‐11T>G is much more pronounced in the minigene experiment than in the patient's lymphocytes where only a small proportion of transcripts is aberrantly spliced (Figure 1). This may be explained by a different composition of *trans*‐acting splicing factors in the transfected cell lines when compared to the patient's blood lymphocytes and/or by lack of *cis*‐acting elements located in intronic sequences or flanking exons that are not contained in the minigene sequence context and that promote exon inclusion even from the mutated sequence in the patient's blood lymphocytes. In any case, this result confirms that c.1722‐11T>G is causative of the splice alterations seen in the patient and, hence, can be considered responsible for the NF1 phenotype of the patient.

We hypothesized that c.1722‐11T>G could affect the 3′ss of intron 15 (11) leading to skipping of exon 16 (12a) with or without exon 15 (11) by two possible mechanisms: (a) replacement of a pyrimidine of the PPT by a purine and/or (b) creation of an AG‐dinucleotide within the AGEZ (Figure 2a). To distinguish between these possible mechanisms, we determined the predicted splice site strength using in silico programs available through Alamut and tested the predictions in minigene experiments. To dissect the two possible mechanisms of action we compared the splice effect of the mutation in the natural sequence context (as present in the patient), where both mechanisms could be at play, with the effect of the mutation in a sequence context where it only replaces a pyrimidine by a purine but does not create an AG‐dinucleotide. That was achieved by introducing a second alteration, c.1722‐12A>G, in cis with mutation c.1722‐11T>A (cf. constructs C2 and C4 in Figure 2a). The c.1722‐12A>G mutation by itself is a splice neutral purine by purine transition which does not substantially alter the predicted splice site strength of the authentic 3′ss of intron 15 (11;Table 1) and has no splicing effect in our minigene experiments (cf. constructs C3 in Figure 2c). According to the splice site prediction programs splice site finder‐like (SSF‐like) and Maximum Entropy (MaxEnt) 1722‐11T>G leads to a substantial reduction of the strength of the authentic 3′ss in the natural context. However, the reduction is much less dramatic when the mutation does not create an AG (cf. splice site prediction scores of wildtype to c.1722‐11T>G, wildtype to c.1722‐12_1722‐11delATinsGG and c.1722‐12A>G to c.1722‐12_1722‐11delATinsGG in Table 1). Our minigene experiments confirmed these predictions. In contrast to the transcripts from the minigene construct C2 containing mutation c.1722‐11T>G, only a small proportion of the transcripts from the minigene construct C4 with the double mutation c.1722‐12_1722‐11delinsGG lack exon 16 (12a) or show inclusion of the last 43 nucleotides of intron 15 (11), particularly when transfected into SH‐SY5Y cells. The latter splice effect results from use of the cryptic 3′ss located 43 nucleotides upstream of the authentic 3′ss (Figure 2a) and is usually observed in the context of a 3′ss mutations affecting the canonical AG‐dinucleotide of intron 15 (11) such as c.1722‐2A>G (see construct C7 in Figure 2c). Differences in the composition of *trans*‐acting splicing factors in the two cell lines may explain why the use of this cryptic 3′ss is more pronounced when the minigene construct C4 is transfected into the SH‐SY5Y cells and exon skipping is more prevalent in the HEK293 cells. Taken together, these results suggest that c.1722‐11T>G exerts its splice effect mainly by creating an AG‐dinucleotide within the AGEZ, which seems to have a stronger impact on the recognition and/or use of the authentic 3′ss than the loss of a pyrimidine of the PPT.

**Table 1:** Scores of the wildtype and mutated intron 15 3′ splice site calculated by five prediction programs

Genotype (name of minigene construct)	wildtype (C1)	c.1722–11T>G (C2)	c.1722‐12 A>G (C3)	c.1722‐12_‐11delATinsGG (C4)	c.1722‐12_1722‐11delATinsTG (C5)	c.1722‐12_1722‐11delATinsTA (C6)	c.1722‐2 A>G (C7)
SpliceSiteFinder‐like	85.36	0	85.36	78.97	84.92	0	0
MaxEntScan	5.54	0	6.73	5.08	5.58	0.18	0
NNSPLICE	0.23	0.05	0.20	0.04	0.27	0.13	0
GeneSplicer	0	0	0	0	0	0	0
Human Splicing Finder	85.60	81.94	85.79	82.13	85.29	85.04	0

*Note*: RefSeq NM_000267.3 is used as reference sequence for reporting the *NF1* variants.

To further ascertain the latter conclusion, we tested whether creation of an AG‐dinucleotide within the AGEZ alone, without concomitant loss of a pyrimidine T within the PPT, would have a similar effect as mutation c.1722‐11T>G. This was modeled by changing c.1722‐11T and c.1722‐12A into c.1722‐12_1722‐11delATinsTA in a minigene construct. This alteration, c.1722‐12_1722‐11delATinsTA, creates an AG‐dinucleotide at almost the same position as the one formed by mutation c.1722‐11T>G, but leaves the total number of pyrimidines and purines in the PPT unchanged (cf. construct C6 in Figure 2a). A similar minigene construct containing variant c.1722‐12_1722‐11delATinsTG served as a control. In this latter construct (C5 in Figure 2a) the purine A of c.1722‐12_1722‐11delATinsTA is replaced by the purine G so that the interchange of nucleotides ‐11 and ‐12 does not create an AG‐dinucleotide. In agreement with the notion that creation of an AG‐dinucleotide within the AGEZ is a major mechanism by which the mutation c.1722‐11T>G leads to loss of the use of the authentic 3′ss, transcripts from construct C6 showed skipping of exon 12a to the same extent as transcripts from constructing C2 while transcripts from construct C5 retained exon 12a just as the wildtype construct C1 (Figure 2c).

### 3.2 Sixty‐three percent of 91 intronic *NF1* 3′ splice site mutations outside the canonical AG‐dinucleotides create an AG‐dinucleotide within the AGEZ and 90% fall into one of three simply defined categories that can be used to select possibly spliceogenic unclassified variants

**Figure 3:**
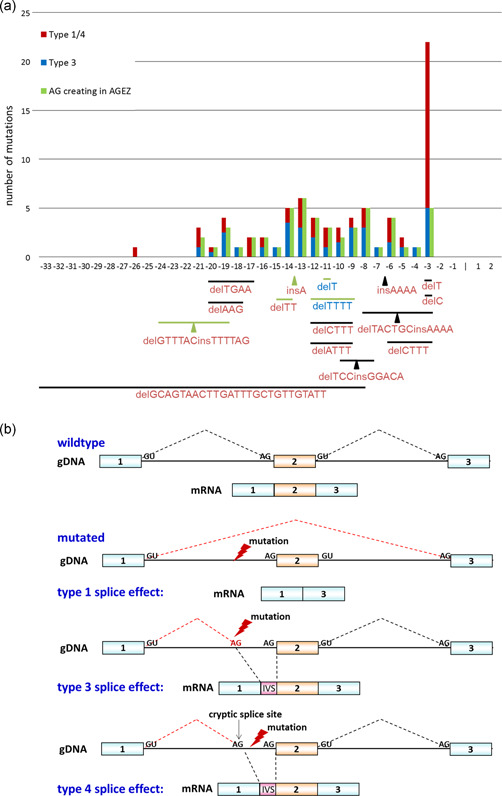
(a) Schematic presentation of 91 *NF1* 3′ splice‐site mutations upstream of the canonical AG‐dinucleotide. Intronic positions ‐33 to ‐1 and the first two nucleotides (1 and 2) of the following exon are indicated on the x‐axis. The noncanonical single nucleotide substitutions are shown in the bar graph. The left bar shows the total number of substitutions per nucleotide position. Mutations that lead to skipping of the downstream exon (type 1) and/or usage of a cryptic 3ʹss (type 4) are displayed in red and mutations that create an AG‐dinucleotide which is used as a novel 3ʹss (type 3) are displayed in blue. The green bars to the right represent the number of these substitutions that generate an AG‐dinucleotide in the AG exclusion zone (AGEZ). Deletions (horizontal lines), delins (horizontal lines and arrowheads) and insertions (arrowheads) are indicated below the bar graph. Lines and arrowheads in green indicate that the mutation generates an AG‐dinucleotide in the AGEZ. The deleted and/or inserted nucleotides are given below the lines and arrowheads. Red letters indicate that the mutation leads to a type 1 or type 4 splice effect and blue letters indicate a type 3 splice effect. (b) Scheme illustrating the type 1, type 4, and type 3 splice effect of intronic 3'ss. Blue and orange boxes indicate exons and the intervening lines intronic sequences of a gene. Wildtype and mutated genomic DNA (gDNA) sequences are shown and the dotted lines indicate which sequences are spliced out to generate the mRNA transcripts as shown below the schematic representation of the gDNA sequences

Prompted by these findings we investigated whether the creation of an AG‐dinucleotide within an AGEZ could be a frequent mechanism of noncanonical intronic 3′ss mutations. To this end, we collected 77 additional *NF1* mutations of this type identified by direct cDNA sequencing in our laboratories and 13 previously reported *NF1* mutations. These 91 mutations (mutation c.1722‐11T>G included) consisted of a total of 75 different single nucleotide substitution variants, 14 different small deletions or deletion‐insertions (delins) of one to 26 nucleotides and two insertions of one and four nucleotides, respectively (Figure 3). These mutations affected nucleotides at positions ‐33 to ‐3 of 38 different *NF1* introns.

According to their splice effect, they fall into three categories: (a) mutations that cause exon skipping of the downstream exon (type 1 splice effect according to (Wimmer et al., 2007)), (b) mutations that lead to utilization of a pre‐existing *cryptic* 3′ss (type 4 splice effect) and (c) mutations that create a novel AG‐dinucleotide which is used as a *novel* 3′ss (type 3 splice effect; Figure 3b). Analyzing mutations affecting nucleotides upstream of position ‐3 (this is at positions ‐33 to ‐4), we noticed that 22 single nucleotide variants (SNVs) including one single nucleotide insertion, and 11 deletions, insertions or delins of more than one nucleotide had a type 1 or 4 splice effect (Table S1 and S2, part A). Twenty‐six SNVs, including one single‐nucleotide deletion, and one 4‐bp deletion affecting all nucleotides at positions ‐21 to ‐4 caused a type 3 splice effect (Tables S1 and S2, part B). Seven SNVs creating novel AG‐dinucleotides, c.289‐6T>G, c.731‐14T>G, c.889‐12T>A, c.5206‐19C>A, c.7000‐10T>G, c.7127‐20T>A, and c.7127‐12T>A, led to skipping of the downstream exon (type 1 splice effect) in a proportion and to the use of the *novel* 3′ss (type 3 splice effect) in the remaining proportion of aberrantly spliced transcripts (listed in part A and part B of Tables S1 and S2). Nineteen of the 24 SNVs, including two single‐nucleotide deletions, at position ‐3 led to a type 1 and/or type 4 splice effect and five to a type 3 splice effect (listed in part C and part D, respectively, of Tables S1 and S2).

Overall, 58 of 91 mutations created an AG‐dinucleotide of which 57/91 (63%) are located within the AGEZ. These 57 mutations include 52/75 SNVs (green bars in Figure 3a) and 4/14 deletions/delins (green lines in Figure 3) and 1/2 single nucleotide insertions (green arrowhead in Figure 3a). As can be deduced from Figure 3 the majority of the 34 mutations which do not create an AG within an AGEZ are either SNV mutations located at position ‐3 of the affected 3′ss (*n* = 19) or deletions/delins/insertions of two or more nucleotides (*n* = 9). Hence, 49/55 (89%) SNVs mutations at positions ‐26 to ‐4, including one single nucleotide deletion and one single nucleotide insertion, created an AG‐dinucleotide within the AGEZ (Table 2, first type of VUS). The novel AG‐dinucleotide is used as the 3′ss AG instead of the authentic 3′ss AG (type 3 splice effect) in 25 of the 49 mutations and 17 of these mutations, including mutation c.1722‐11T>G, lead to skipping of the downstream exon (type 1 splice effect) and/or use of a cryptic exonic or intronic 3′ss (type 4 splice effect). Seven mutations (listed above) have a type 3 and a type 1 splice effect, each only in a proportion of the aberrantly spliced transcripts.

**Table 2:** Criteria to test intronic VUS at 3′ splice site for a splice effect as deduced from 91 *NF1* mutations

Type of VUS	Criteria of VUS	Criteria fulfilled (no. of VUS)		Total no. of VUS in category
		yes	no	
SNV upstream of position ‐3	creates AG in AGEZ	49	6	55
SNV at position ‐3	Y>R	23	1	24
del, ins, delins of>1 nt	removes ≥ 2 Y from AGEZ or creates AG in AGEZ	9	3	12
	Total no. of VUS	81	10	91

Abbreviations; AGEZ, AG exclusion zone; del, deletion; dup, duplication; ins, insertion; nt, nucleotide; SNV, single nucleotide variant (including single nucleotide deletions, duplications, and insertions); VUS, variants of unknown significance; R, purine; Y, pyrimidine.

Of the six SNV mutations at positions ‐26 to ‐4 not creating an AG‐dinucleotide within the AGEZ, three affect a predicted BPS: c.1722‐26T>C (intron 15 (11)) and c.4515‐19A>G and 4515‐21T>C (both intron 34 (26)). Mutation c.4515‐19A>G, which also forms a novel 3′ss, but sits outside the AGEZ of the authentic 3′ss, causes a type 3 splice effect. In contrast, mutation c.4515‐21T>C, which only affects the potential BPS but does not create an AG‐dinucleotide, leads to the use of two intronic cryptic 3′ss (type 4 splice effect) which are located 14 and 17 nucleotides upstream of the authentic 3′ss. Mutation c.1722‐26T>C also leads to use of a cryptic 3′ss 43 nucleotides upstream of the authentic 3′ss. The mutations, 6642‐5T>G, 6757‐10T>G, and 6757‐9T>G, are the only three SNV mutations located within the AGEZ and upstream of position ‐3 which do not create an AG‐dinucleotide. Including these three mutations, 26/27 single nucleotide substitutions within the AGEZ that are associated with a type 1 or 4 splices effect replace a pyrimidine by a purine, thereby weakening the predicted strength of the PPT. In contrast, only 13/24 single nucleotide substitutions associated only with a type 3 splice effect and located upstream of position ‐3 within the AGEZ replace a pyrimidine by a purine (Table S2).

Taken together, these results suggest that the creation of an AG within the AGEZ accounts at least partly for the splice effect of 89% (49/55) of SNV mutations, including single‐nucleotide deletions and insertions, upstream of position ‐3 (Table 2, the first type of VUS). Other mechanisms may be responsible for the observed splice effects of mutations at position ‐3 and deletions/insertions of more than one nucleotide. Only 5/24 SNVs, including two single‐nucleotide deletions, at position ‐3 create a tandem AG‐AG of which the newly created AG, which is more proximal to the BP, is used (Tables S1 and S2, part D). However, all but one SNV at this position replace a pyrimidine by a purine (Table 2, the second type of VUS). This is in line with the notion that a pyrimidine (C or T) at position ‐3, which is found in 96% of all mammalian 3′ss (Shapiro & Senapathy, 1987; Zhang, 1998), is crucial for their recognition and/or use by the splicing machinery.

Eight of the 11 deletions and delins of two or more nucleotides lead to loss of at least two pyrimidines from the PPT, suggesting that they exert their splice effect by affecting the “strength” of the PPT (Table 2, a third type of VUS). One delins, c.7807‐24_7807‐19delGTTTACinsTTTTAG, not altering the number of pyrimidines creates an AG in the AGEZ (listed as positive in Table 2, third type of VUS). This delins is also affecting a predicted BPS as do two other multi‐nucleotide deletions, c.1722‐20_1722‐17delTGAA and c.4515‐20_4515‐18delAAG, which are listed as not fulfilling the criteria (Table 2, third type of VUS) because they do not create an AG in the AGEZ nor lead to loss of >1 pyrimidine from the AGEZ. The only insertion of >1 nucleotide, c.6642‐7_6642‐6insAAAA, introduces four purines in the AGEZ/PPT (conservatively listed as negative in Table 2, third line).

In conclusion, 81 (89%) of the 91 intronic 3′ss mutations outside the canonical AG‐dinucleotide fall into one or more of the following categories: (a) variant that creates an AG within the AGEZ; (b) variant at position ‐3 that replaces a pyrimidine (Y) by a purine (R); (c) variant (mainly multi‐nucleotide deletions) that removes two or more pyrimidines from the PPT/AGEZ (Table 2). These categories could be used as criteria to select among unclassified variants, which are identified by a gDNA‐based mutation analysis protocol, those which are likely to alter splicing. The suggested rule would be that any variant at the 3′ ends of introns fulfilling one or more of these criteria should be further evaluated for a possible splice effect.

### 3.3 The three newly defined selection criteria for noncanonical intronic 3′ splice site mutations are highly sensitive and specific in a validation cohort of 110 variants

To further evaluate the proposed selection criteria for their sensitivity and specificity we analyzed 48 *BRCA1*, 46 *BRCA2*, 12 *CFTR*, and each 2 *CTRC* and *RHD* intronic variants at the 3′ss (position ‐113 to ‐3; Supp. table S4) that were collected by the French Unicancer Group (UGG) and included in a larger cohort of other variants with and without splice effect which was used to define guidelines for splicing analysis in molecular diagnosis (Houdayer et al., 2012; Leman et al., 2018). These 110 variants included 34 with a reported splice effect and 76 without splice effect. Of the variants reported to have a splice effect, 26/34 fulfill one of the proposed selection criteria, versus only 8/76 without splice effect, (Table S4). Hence, from this variant data‐set a sensitivity and specificity of 76% and 89%, respectively, as well as a positive predictive value (PPV) of 76% and negative predictive value (NPV) of 89%, would be deduced for the proposed selection criteria. With 8 false negatives and 8 false positives of 110 predictions the accuracy of the proposed selection criteria is 85% in this cohort.

## 4 DISCUSSION

Impact of our findings on the understanding of the molecular mechanism of 3′ss recognition and selection:

Splicing in general and, in particular, the processes involved in the recognition and selection of 3′ss are complex and still poorly understood. Uncovering motives of action of splice mutations is one way to unravel the molecular mechanisms by which pathogenic variants impair splicing which will ultimately lead to a better understanding of the process.

Through the in‐depth analysis of the *NF1* variant c.1722‐11T>G and the subsequent evaluation of 91 disease‐causing *NF1* variants located at the 3′end of an intron but outside the canonical AG‐dinucleotide we show here that creation of a novel AG within the AGEZ, that is the intronic sequence between the BP and the AG of the *authentic* 3′ss, is a frequent action motive of intronic sequence variants with a splice effect. A novel AG in the AGEZ was created by 57/91 (63%) of all mutations and 49/55 (89%) of the SNV (including single‐nucleotide deletions and insertions) upstream of position ‐3. Overall, this number is comparable to the 86% (62/72) of intronic mutations outside the 3′YAG that were found by Vorechovsky (Vorechovsky, 2006) to create AGs. However, in contrast to Vorechovsky's findings, according to which 93% (58/62) of these AGs were used as *novel* 3′ss in vivo (Vorechovsky, 2006), only two thirds (32/49) of the novel AGs analyzed in our study are used in all (*n* = 25) or at least in a proportion (*n* = 7) of the aberrantly spliced transcripts as *novel* 3′ss instead of the *authentic* 3′ss (Type 3 splice effect). This difference results from ascertainment bias in Vorechovsky's mutation cohort which consisted only of variants that either activated a cryptic 3′ss or were used as a “de novo” 3′ss but did not include variants leading to exon skipping. Overall, the view that the *novel* 3′ss appear to out‐compete the *authentic* 3′ss in the mutated sequence context is reflected when comparing the scores of the *novel* 3′ss with the scores of the *authentic* 3′ss in the mutated sequence context using the programs Max‐EntScan (MES; Yeo & Burge, 2004) and splice site finder (SSF) (Shapiro & Senapathy, 1987; Zhang, 1998). The mean scores of the *novel* 3′ss are substantially higher than the mean score of the *authentic* 3′ss in the mutated sequence context where it is substantially lower than in the wildtype context (see Table 3 and Table S2). However, there are exceptions to this general rule, especially when the newly generated AG‐dinucleotide is located upstream of position ‐12 in the intron, that is upstream of the splice consensus sites described by Cartegni, Chew, and Krainer (2002) (see e.g., 2410‐15A>G, 2410‐14A>G in Table S2). Furthermore, the mean score of the *novel* 3′ss is lower than the mean score of the *authentic* 3′ss in the wildtype context. The main question arising from this finding is therefore by which molecular mechanisms the newly generated AG‐dinucleotide interferes with the use of the *authentic* 3′ss which leads than either to the use of the variant‐generated AG‐dinucleotide as *novel* 3′ss (type 3 effect) or in other instances, to exon skipping or use of a pre‐existing *cryptic* 3′ss that is different from the newly generated AG‐dinucleotide (type 1 or type 4 effect, Figure 3b). The model of a linear scanning mechanism that selects the first AG downstream of the BP in the second catalytic step of mRNA splicing (Umen & Guthrie, 1995) may be applicable to a type 3 splice effect. However, it is not applicable to type 1 and type 4 effects resulting from almost 50% of the 49 mutations either in all (*n* = 17) or a proportion of the aberrantly spliced transcripts (*n* = 7). As most (22/24) newly created AGs that lead to a type 1 or 4 splice effect are Y>R changes, they may act through decreasing the affinity of the PPT to U2AF65. However, using mutation c.1722‐11T>G as an example our minigene experiments clearly show that the creation of a novel AG in the AGEZ is a strong action motive for these intronic 3′ splice sites, as well.

**Table 3:** Mean of the 3′ splice site scores calculated for the *authentic* and *novel* 3′ splice sites (3′ss) by MaxEntScan (MES) and splice site finder (SSF)

Splice effect of the mutation (number of mutations)	*authentic* 3′ss (wildtype sequence)	*authentic* 3′ss (mutated sequence)	*novel* 3′ss (mutated sequence)
	Mean score calculate by MES		
type 3 effect (*n* = 32)	6.83	2.79	5.55
type 1/4 effect (*n* = 24)	7.92	3.87	3.60
	Mean score calculate by SSF		
type 3 effect (*n* = 32)	82.78	27.32	77.65
type 1/4 effect (*n* = 24)	88.52	31.74	66.58

One possible explanation applicable to both types of splice effects arises from a study of Chen et al. (2017) which was aimed at defining the pathways by which the key components involved in the early steps of splicing recognize and select candidate 3′ss sites. Their data suggest that there is no intrinsic process that leads to a single U2AF binding to one pre‐defined authentic 3′ss, instead they suggest that in the early stages of splicing (complex E) a number of candidate 3′ss located in close vicinity to each other can be bound by the U2AF35 subunit. However, concurrent occupancy of candidate 3′ss leads to slower splicing. Chen et al. suggest a model where sur‐plus U2AF35 bound outside the authentic 3′ss need to be removed to allow the formation first of an intermediate complex (complex I) followed by an active splice complex (complex A). If bound sur‐plus U2AF35 resist displacement, they compromise complex A formation and use of any of the U2AF35 bound 3′ss. Following this model, generation of an AG within the AGEZ could lead to multiple 3′ss candidate sites bound by a U2AF35 subunit which can lead either to displacement of U2AF35 from the *authentic* 3′ss and complex A formation using the *novel* U2AF35 bound 3′ss (type 3 splice effect) or to no formation of an active splice complex at any of the U2AF35 bound 3′ss candidate sites located in close vicinity and leading to a type 1 or type 4 splice effect. Alternatively, or in addition, it is conceivable that U2AF35 binding at the mutation‐generated AG in the AGEZ could sterically interfere with the recognition of the PPT by U2AF65, and, consequently the formation of the U2AF heterodimer which is essential for U2 snRNP recruitment to the BPS and complexes A formation. Given that the 3′ss motive YAG is also contained in the recognition motive of the splicing repressor hnRNP A1 (Bruun et al., 2016), it may also be speculated that the AG‐creating mutations in the AGEZ could exert their effect on splicing by creating an hnRNPA1 binding motif. The observation that the majority of *NF1* mutations (40/53) generating an AG in the AGEZ upstream of position ‐3 are preceded by a C or T and, therefore, fit with the 3′ss motive YAG also contained in the hnRNP A1 recognition motif, may be taken as a support for these hypotheses. Nevertheless, it should be noted that five of the *NF1* SNVs with a type 1 and six with a type 3 splice effect create an AAG or GAG in the AGEZ upstream of position ‐3 (see Table S2). Furthermore, a minigene construct containing the *NF1* variant c.1722‐11T>A creating an AAG in the AGEZ exerts a strong splice effect similar to the splice effect of mutation c.1722‐11T>G, which creates a TAG (Figure S2).

Hence, although the exact molecular mechanisms of action remain to be understood, our results suggest that absence of AG‐dinucleotides in the region between the BP and the AG of an authentic 3′ss may not only be necessary to ensure accurate AG selection during the catalytic Step II of splicing but also for appropriate assembly of the splicing components in the initial steps of 3′ss recognition and selection. Our findings may render new avenues to further study this model.

Two other frequent action motives that were identified in this study are removal or replacement by purines of >1 pyrimidine from the PPT/AGEZ and replacement of a pyrimidine at position ‐3 of the 3′ss by a purine. The most conceivable molecular mechanisms by which mutations following these motives interfere with the selection and use of the *authentic* 3′ss is by decreasing the affinity of the PPT and the 3′ss YAG to the U2AF65 and U2AF35 subunits, respectively.

Impact of our findings on mutation analysis in clinical and research settings:

Independent from the question by which molecular mechanisms the analyzed intronic splice mutations impair the recognition and selection of the *authentic* 3′ss and lead to aberrant splicing, the identification of three motives of action of noncanonical 3′ss mutations will be clinically highly useful.

With the increased use of massively parallel sequencing technologies, enabling the more widespread use of gene panel and exome sequencing in clinical molecular diagnostics, the number of VUS, in exons and introns has increased substantially. The way intronic VUS may impact the functionality of the encoded protein is by altering splicing. A potential splice effect of an intronic VUS is preferentially confirmed in mRNA isolated from cells of the patient. Minigene experiments will be needed if appropriate cells expressing the gene are not available. Both types of experiments are labor‐intensive and less automatable than massive parallel sequencing. Hence, given the high number of intronic VUS, it is routinely not feasible to assess a potential splice effect of all identified VUS and a selection of those most likely to have a splice effect has to be made.

From our analysis of noncanonical 3′ splice site mutations we deduce three simple rules to select VUS at the 3′ end of an intron for further splice analyses: Select all VUS (a) that create an AG within the AGEZ, (b) that replace a pyrimidine (Y) by a purine (R) at position ‐3, and/or (c) that remove >1 pyrimidine from the PPT/AGEZ. According to our results in 91 bona fide *NF1* splice mutations, these criteria have a sensitivity of 89% and from the analysis of 110 variants in the *BRCA* and other genes, we would calculate an accuracy of these criteria of 85% (94/110 correctly predicted), an NPV of 89% and a PPV of 76%. Of note, the sensitivity of these criteria was lower in the validation cohort than in the cohort of *NF1* mutations (76% vs. 89%). However, the validation cohort contains a smaller number (*n* = 34) of variants with a splice effect and splicing was not assessed uniformly by transcript analysis (as was done in our laboratories) controlled for NMD in patient cells, but with several different methods. This may impact the accuracy of these calculations in the validation cohort.

The most widely used methods to predict a potential splice effect of a VUS rely on the use of freely available programs calculating the “strength” of splice sites. Based on the analysis of 272 *BRCA1* and *BRCA2* variants with and without splice effects Houdayer and colleagues developed a strategy to use the programs MaxEntScan (MES; Yeo & Burge, 2004) and splice site finder (SSF; Shapiro & Senapathy, 1987; Zhang, 1998) as made available through the Alamut Visual software to predict whether a VUS is likely or unlikely to affect splicing (Houdayer et al., 2012). The suggested rule is to select any VUS with a mutant score of the authentic splice site that is 15% lower than the wildtype score of this authentic splice site when calculated with the program MES and also 5% lower than the wildtype score when calculated with SSF. This rule works with high sensitivity and specificity for VUS located at the splice site consensus sites described by Cartegni et al. (2002), but is not reliable outside these consensus sites. For the 3′ss this consensus sequence spans position ‐12 in the intron to +2 in the exon. Using logistic regression analysis on a larger data‐set this strategy was further developed into a bioinformatics program named *Splicing Prediction in Consensus Elements* (SPiCE) that generates scores (Leman et al., 2018). Optimal sensitivity and specificity of splice effect prediction are reached on the analyzed variants when the thresholds were set on SPiCE scores of 0.115 and 0.749, respectively, with scores <0.115 predicted not to affect splicing and scores >0.749 predicted to affect splicing. About 10% of the analyzed variants fall into the “gray area” in‐between these thresholds. Calculating the SPiCE scores for 60 of our 91 noncanonical *NF1* splice mutations, which are located within the defined 3′ss consensus site, two are false negatives (score <0.115) and three fall into the “gray area” while the remaining 55 have scores >0.749. Hence, depending on whether the threshold for further analyses is set at 0.115 or 0.749, the SPiCE program reaches a sensitivity of 97% (58/60) or 92% (55/60). Of note, four of the five *NF1* splice mutations with a score <0.749, that is c.4515‐3delT, c.6642‐5T>G, c.6757‐10T>G and c.6757‐9T>G, also belong to the 5/60 mutations in this cohort that do not fall into one of the here proposed “actionable” motives/selection criteria for noncanonical 3′ss mutations. The fifth mutation with a SPiCE score <0.115, c.2851‐6_2851‐3delCTTT, falls into the category of variants removing two or more pyrimidines from the PPT. Vice versa, the SPiCE score of 1.00 for mutation c.6642‐7_6642‐6insAAAA clearly predicts a splice effect of this mutation inserting four purines into the PPT, which is a rare mutation type that we did not define as a separate selection criteria.

Taken together, the three here defined action motives by which noncanonical 3'ss mutations exert their splice effect are well recognized by the splice site prediction tools MES and SSF, and consequently the SPiCE program. Hence, the selection of potentially splicogenic VUS will be highly sensitive and specific when applying for the SPiCE program as well as when applying the here defined selection criteria, but both approaches will have difficulties with variants that do not fall into one of the here identified action motives.

The major advantage of using the here defined selection criteria is their applicability also to variants located upstream of the position ‐12 where the SPiCE program is not applicable. Roughly a third (31/91) of all bona fide *NF1* splice mutations affect nucleotides in this intronic region. 26 of these 31 mutations (84%) fall into one of the three action motives we identified and would, therefore, have been selected as potentially spliceogenic by the here defined selection criteria.

## CONFLICT OF INTERESTS

The authors declare that there are no conflict of interests.

## Supporting information

Supporting information

Supporting information

## Data Availability

The data that support the findings of this study are available from the corresponding author upon reasonable request.
