# Supplementary material for: AG‐exclusion zone revisited: Lessons to learn from 91 intronic NF1 3′ splice site mutations outside the canonical AG‐dinucleotides
Source: Hum Mutat. 2020 Mar 11;41(6):1145–56. doi: 10.1002/humu.24005 (PMC7317903; doi:10.1002/humu.24005)
Supplement: Supplementary file 2 — Supporting information [file HUMU-41-1145-s002.docx]

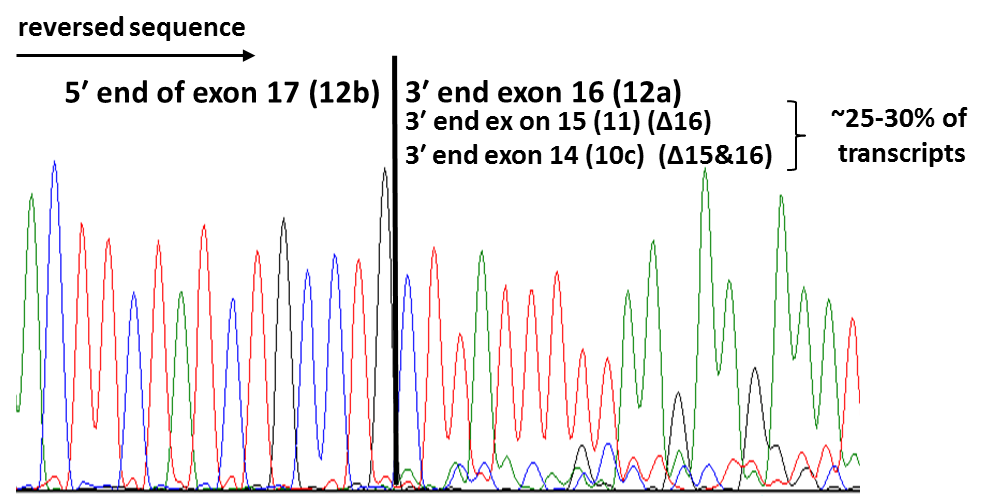


**Supp. Figure S1:** Direct cDNA sequencing of patient F8519 carrying mutation NF1 c.1722-11T>G reveals aberrantly spliced transcripts. Sanger sequence (antisense direction) of transcripts isolated from puromycin-treated short-term lymphocyte cultures of the patient at the border of exon 17 (12b) and exon 16 (ex 12a). Two back ground sequences are visible which come from approximately 25-30% of transcripts that either lack exon 16 (12a) only (∆16) or exons 15 (11) and 16 (12a) (∆15&16).


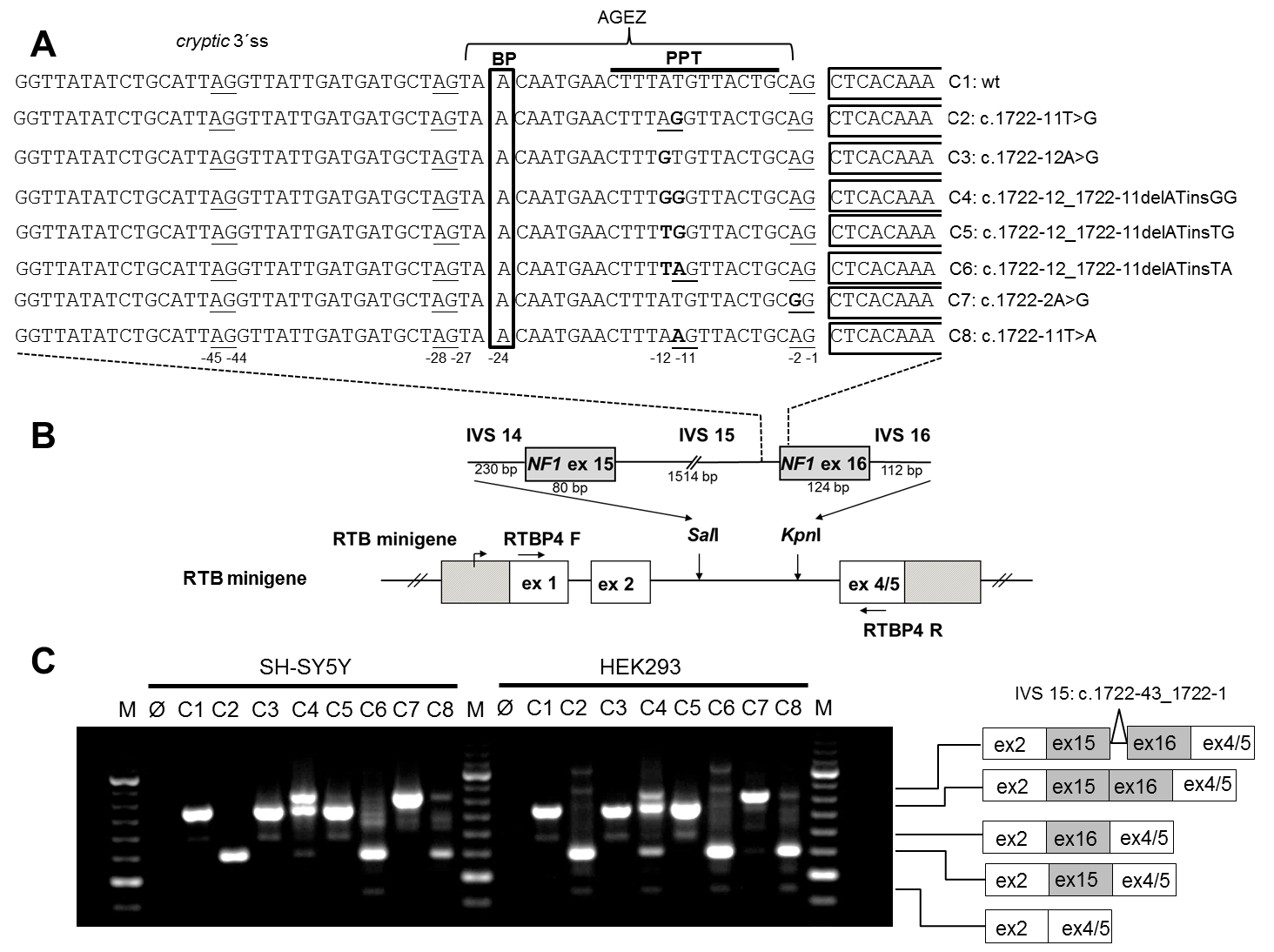


**Supp. Figure S2:** Minigene experiments confirming that the *NF1* variant c.1722-11T>A has a similar splice effect as *NF1* mutation c.1722-11T>G and reveal that the mutation acts mainly by creation of an AG-dinucleotide within the AG exclusion zone. (A) Wildtype (wt) and mutated sequences of the intron 15 (IVS 15) 3' splice site (3'ss) which are contained in the RTB minigene, constructs C1-C8, are shown. The first nucleotides of exon 16 (ex 16) are framed. The mutated nucleotides are in bold letters. The adenine of the predicted branch point (BP), the polypyrimidine tract (PPT) and a cryptic 3'ss are indicated. All AG-dinucleotides in the intron are underlined and the AG exclusion zone (AGEZ) is indicated. (B) Schematic diagram of RTB minigene and the inserted *NF1* sequences containing wildtype and mutated IVS 15 and the flanking exons 15 and 16 with flanking intronic sequences. The positions of primers RTBP4F and RTBP4R used for RT-PCR are indicated. (C) RT-PCR results of mRNA isolated from transient transfections of SH-Sy5y and HEK293 with minigene constructs C1-C8. The positions of the PCR products from correctly and aberrantly spliced transcripts are indicated on the left. Use of the cryptic 3'ss leads to insertion of the last 43 nucleotides of IVS15 (c.1722-43_1722-1).
